# Metabolomic signatures for visceral adiposity and dysglycaemia in Asian Chinese and Caucasian European adults: the cross-sectional TOFI_Asia study

**DOI:** 10.1186/s12986-020-00518-z

**Published:** 2020-11-16

**Authors:** Zhanxuan E. Wu, Karl Fraser, Marlena C. Kruger, Ivana R. Sequeira, Wilson Yip, Louise W. Lu, Lindsay D. Plank, Rinki Murphy, Garth J. S. Cooper, Jean-Charles Martin, Sally D. Poppitt

**Affiliations:** 1 Food Nutrition and Health, Food and Bio-Based Products, AgResearch Limited, Palmerston North, 4442 New Zealand; 2 School of Health Sciences, Massey University, Palmerston North, 4442 New Zealand; 3 High-Value Nutrition National Science Challenge, Auckland, New Zealand; 4 Riddet Institute, Massey University, Palmerston North, 4442 New Zealand; 5 Human Nutrition Unit, School of Biological Sciences, University of Auckland, Auckland, 1010 New Zealand; 6 Department of Surgery, University of Auckland, Auckland, 1010 New Zealand; 7 Department of Medicine, School of Biological Sciences, University of Auckland, Auckland, 1010 New Zealand; 8 Centre for Advanced Discovery and Experimental Therapeutics, School of Medical Sciences, Faculty of Biology, Medicine and Health, University of Manchester, Manchester, M13 9NT UK; 9 INSERM, INRA, C2VN, BioMeT, Aix-Marseille University, Marseille, France

**Keywords:** Metabolomics, Type 2 diabetes, Visceral adiposity

## Abstract

**Background:**

Asian Chinese are more susceptible to deposition of visceral adipose tissue (VAT) and type 2 diabetes (T2D) development than European Caucasians when matched for gender, age and body mass index (BMI). Our aims were: (i) characterise the ethnicity-specific metabolomic signature of visceral adiposity measured by dual energy X-ray absorptiometry (DXA) and fasting plasma glucose (FPG), and (ii) identify individuals susceptible to worse metabolic health outcomes.

**Methods:**

Fasting plasma samples from normoglycaemic (n = 274) and prediabetic (n = 83) participants were analysed with liquid chromatography–mass spectrometry using untargeted metabolomics. Multiple linear regression adjusting for age, gender and BMI was performed to identify metabolites associated with FPG and VAT calculated as percentage of total body fat (%VAT_TBF_) in each ethnic group. Metabolic risk groups in each ethnicity were stratified based on the joint metabolomic signature for FPG and %VAT_TBF_ and clinically characterised using partial least squares-discriminant analysis (PLS-DA) and t-tests.

**Results:**

FPG was correlated with 40 and 110 metabolites in Caucasians and Chinese respectively, with diglyceride DG(38:5) (adjusted β = 0.29, *p* = 3.00E−05) in Caucasians and triglyceride TG(54:4) (adjusted β = 0.28, *p* = 2.02E−07) in Chinese being the most significantly correlated metabolite based on the p-value. %VAT_TBF_ was correlated with 85 and 119 metabolites in Caucasians and Chinese respectively, with TG(56:2) (adjusted β = 0.3, *p* = 8.25E−09) in Caucasians and TG(58:3) (adjusted β = 0.25, *p* = 2.34E−08) in Chinese being the most significantly correlated. 24 metabolites associated with FPG were common to both ethnicities including glycerolipid species. 67 metabolites associated with %VAT_TBF_ were common to both ethnicities including positive correlations with dihydroceramide, sphingomyelin, glycerolipid, phosphatidylcholine, phosphatidylethnolamine, and inverse correlations with ether-linked phosphatidylcholine. Participant re-stratification found greater total and central adiposity, worse clinical lipid profiles, higher serum glucoregulatory peptides and liver enzymes in normal fasting glucose (NFG) individuals with a prediabetic metabolomic profile than NFG individuals with a normoglycaemic metabolomic profile in both ethnicities.

**Conclusions:**

Untargeted metabolomics identified common and disparate metabolites associated with FPG and %VAT_TBF_, with an ethnic-dimorphic signature for these metabolic traits. These signatures could improve risk stratification and identify NFG individuals with an adverse cardiometabolic and T2D risk profile.

## Background

The prevalence of type 2 diabetes (T2D) has witnessed a drastic surge in China over recent decades. There are now 110 million people with T2D in China [1], reaching epidemic proportions and predicted to continue on this trajectory [2]. Whilst the accelerated rise of the T2D prevalence is likely due to a rapid transition to a westernised diet and lifestyle [3, 4], striking observations of younger and outwardly slimmer individuals with diabetes in South and East Asia highlights the role of ethnicity in T2D risk [5]. Although high BMI has been a well-established risk factor for T2D [6], it is evident that even modest weight gain greatly increases T2D risk in Asians compared to modest increases in other ethnicities [7]. The mechanisms underpinning the greater risk of poor metabolic health in Asian populations have not yet been elucidated, but it is clear that site of fat deposition plays an important role [8–10], and Asian populations are more prone to abdominal and visceral adiposity as well as ectopic fat deposition into key organs including pancreas and liver [9, 11, 12]. Excess visceral adipose tissue (VAT) and ectopic fat deposition are associated with higher risks of cardiometabolic diseases and complications [13–15]. It has been hypothesised that poor adipose expandability in ‘safer’ subcutaneous sites may promote this ‘lipid overspill’ and ectopic storage [16–18]. The TOFI (Thin-on-the-Outside-Fat-on-the-Inside) profile has been coined for these individuals who may have hidden risks of cardiometabolic disease [19].

Current measurements of VAT and ectopic organ fat rely on imaging techniques which are expensive and time-consuming. Whilst blood glucose and HbA_1c_ both provide simple and cost-effective markers of prediabetes, predicting individuals who remain in the prediabetic state for many years vs those progressing rapidly to T2D is difficult from these single markers. Metabolomics provides a useful means to advance the understanding of disease pathophysiology, and may facilitate the identification of at-risk individuals before dysglycaemia occurs and/or predict those who will rapidly worsen to T2D [20, 21]. For example, metabolic shifts in branched-chain amino acids (BCAAs) are the most prominent changes associated with insulin resistance (IR) and predictive of future T2D development to date [22]. Increased levels of circulating BCAAs can be due to increased protein degradation in IR [23], while evidence for higher levels of C3- and C5-acylcarnitine, the catabolic intermediates of BCAAs, associated with IR and T2D have also been reported, suggesting that higher levels of circulating BCAA may also be due to an impaired catabolism resulting from mitochondrial overload and metabolic inflexibility [24]. Higher levels of medium-chain acylcarnitines (MCAC) were reported in T2D patients, which has been postulated as a consequence of incomplete fatty acid oxidation (FAO) [25]. However, higher levels of long-chain AC (LCAC), which may be indicative of an excessive lipid load and FAO flux, were found to be predictive of incident T2D [26]. Mechanistic investigation suggested accumulation of LCAC and MCAC was due to mitochondrial overload and discordant FAO and citric acid cycle (TCA) activity [27, 28]. Other metabolites such as those involved in sugar metabolism, purine metabolism and the urea cycle have been occasionally reported [29], and the identification and understanding of metabolite markers for T2D is ongoing. There are few studies investigating the metabolomic profile associated with increased VAT. To date, several AAs, organic acids, ether-linked phosphatidylcholines (PCs), lysoPCs-to-PCs ratio have been reported as markers for VAT [30–33].

Given that increased VAT deposition is a risk factor for T2D development and may underlie an increased propensity for poor metabolic health in specific populations such as Asians, a major gap in the field remains as to how characteristic plasma signatures for VAT and glycaemia are related to each other, and whether the metabolite markers for these two metabolic traits are common or unique to different ethnic groups. Herein, we simultaneously profiled the plasma samples from non-diabetic individuals across a range of FPG and VAT content calculated as percentage of total body fat (%VAT_TBF_) from 2 ethnic groups (Asian Chinese and age- and BMI-matched Caucasian Europeans). FPG was focused on in this study as it is one of the 3 measurements (along with HbA1c and oral glucose tolerance test) for prediabetes/T2D diagnosis according to the American diabetes association (ADA) criteria [34]. FPG and HbA1c are convenient and less time-consuming to obtain thus are suitable for large-scale studies. The level of FPG is less subject to haemoglobin variants or certain conditions e.g. sickle cell disease or recent blood transfusion as opposed to HbA1c [34], hence was chosen as an outcome variable to be investigated in this study. The goals of this study were (1) to determine whether plasma metabolomic profiles measured using an unbiased untargeted approach differed between ethnicities; (2) to characterise the metabolomic signatures associated with %VAT_TBF_ and FPG in each ethnic group; (3) to predict FPG state using the metabolomic signature (i.e. metabolic FPG state) and characterise the clinical profiles of the metabolic FPG state.

## Methods

### Study cohort

The TOFI-Asia study is a cross-sectional study conducted at the Human Nutrition Unit (HNU), University of Auckland, New Zealand. All participants self-reported both parents of the same ethnic descent, i.e. European Caucasian or Asian Chinese according to ethnic group profiles by Stats New Zealand, Tatauranga Aotearoa) [35]. Participants were recruited for both genders across a wide range of ages (20–70 years) and BMI (20–45 kg/m^2^), and were either normoglycaemic or prediabetic based on ADA criteria [36]. They had no significant weight gain or loss (> 10%) in the previous 3 months, no prior bariatric surgery, were not pregnant, breastfeeding, or currently taking glucose-related medications (e.g. glucocorticoids) or had a significant current or prior history of disease including T2D. A detailed description of the study population and protocol can be found elsewhere [37]. In total, 199 Asian Chinese and 158 European Caucasian participants were enrolled in the study. All participants attended the study visit following an overnight fast.

### Phenotypic Characterisation and Laboratory Measurements

Body weight, height, waist and hip circumference, and blood pressure were recorded at HNU. Fasting venous blood samples were collected, separated, and stored at −80 °C until analysis. FPG was measured using the hexokinase method. HbA_1c_ was determined by capillary electrophoresis. Plasma glucoregulatory peptides (insulin, C-peptide, glucagon, total amylin, gastric inhibitory polypeptide (GIP) and glucagon-like peptide-1 (GLP-1)) were analysed by multiplex immunoassay. Serum liver enzymes (alanine transaminase (ALT), aspartate transaminase (AST), alkaline phosphatase (ALP), gamma-glutamyltransferase (GGT)) and lipids (total cholesterol, total triglyceride (TG), HDL-cholesterol) were analysed using internationally accredited methods. Details of sampling procedures and blood measurements have been reported elsewhere [37].

### Body composition

Dual energy X-ray absorptiometry (DXA) (iDXA, GE Healthcare, WI, USA) scanning was used to obtain total body fat (TBF), abdominal adipose tissue (AAT) and VAT mass as previously described [37]. One participant did not attend DXA scanning therefore 356 body composition profiles were available.

Region-specific percentage measures were obtained;

$$\begin{aligned} & \% {\text{TBF }} = { 1}00 \% \, \times {\text{ TBF mass}}/\left( {{\text{total body lean mass }} + {\text{ TBF mass}}} \right); \\ & \% {\text{AAT }} = { 1}00 \% \, \times {\text{ AAT mass}}/\left( {{\text{abdominal lean mass }} + {\text{ AAT mass}}} \right) \\ \end{aligned}$$

Visceral adipose tissue was calculated in 2 ways:

i %VAT_TBF_ = 100% × VAT mass / TBF mass
j %VAT_AAT_ = 100% × VAT mass /AAT mass

%VAT_TBF_ is used as a measure of visceral adiposity in this manuscript and the dependent variable to be characterise by metabolomics. This is because it considers both total body fat mass (i.e. outside the viscera) and VAT mass (i.e. inside the viscera), and hence is representative of the “TOFI” phenotype of interest to the present study.

### Sample preparation for LC–MS untargeted metabolomics

Samples were randomised into 4 batches and extracted on 4 consecutive days. The protocol for metabolite extraction was adapted from a previously reported method [38]. Briefly, 100 µL plasma was mixed with 800 µL pre-chilled (−20 °C) CHCl_3_:MeOH (50:50, v/v) containing internal standard compounds (detail provided in Additional file 2: Table S1), agitated for 30 s and placed in a −20 °C freezer for 60 min to allow protein precipitation, followed by addition of 400 µL H_2_0, vortex-mixing for 30 s and centrifugation (Eppendorf Centrifuge 5427 R, Eppendorf, Hamburg, Germany). Centrifuge parameters were set at 11,000 rpm, 4 °C, 10 min. Two blank samples were prepared following the same protocol replacing plasma with H_2_0. 200 µL of the upper aqueous layer and 200 µL of the lower organic layer were transferred into two 2 mL microcentrifuge tubes separately, dried down under a nitrogen stream and stored at −80 °C. To account for intra- and inter-batch variation, pooled QC samples were prepared by combining an aliquot of the upper or lower phase from every sample extracted on the same day in a clean glass tube and stored at −80 °C. At the end of all sample extractions, the pooled samples on each day were then combined, dispensed into separate 200 µl aliquots and dried down under the nitrogen stream and stored at −80 °C. On the day of instrumental analysis, dried polar and lipid extracts were reconstituted in 200 µL acetonitrile:H_2_O (50:50, v/v) and modified Folch solution (CHCl_3_:MeOH:H_2_O, 66:33:1, v/v/v) containing pre-dissolved 0.01% 16:0 d_31_-18:1-PE internal standard [0.01% (%w/v)] for polar metabolite and lipid analysis respectively. The reconstitution volume was determined using a previously described workflow [39].

### Ultra-performance liquid chromatography (UPLC)-mass spectrometry analysis of lipids

Lipid analyses were performed using an Accela 1250 quaternary UHPLC system coupled to a Q Exactive hybrid quadrupole-Orbitrap mass spectrometer (Thermo Fisher Scientific, Waltham, MA, USA) with a heated electrospray ionisation source set to 370 °C. An Acquity CSH™ C18 column 1.7 µm, 2.1 mm × 100 mm (Waters, Milford, MA, USA) was used for lipid separation with a column temperature of 65 °C and mobile phase flow rate at 600 µL/min. The mobile phases consisted of acetonitrile/H_2_O (60:40) with 10 mM ammonium formate and 0.1% formic acid (A), and isopropanol/acetonitrile (90:10) with 10 mM ammonium formate and 0.1% formic acid (B). Analytes were eluted from the column with the following gradient program: 15–30% B (0.0–2.0 min), 30–48% B (2.0–2.5 min), 48–82% B (2.5–11.0 min), 82–99% B (11.0–11.5 min), 99% B was maintained for 3.5 min followed by re-equilibration with 15% B for 3 min. Two microliter reconstituted samples were injected. External mass calibration of the Orbitrap prior to sample analysis was performed by flow injection of the calibration mix solution according to the manufacturer’s instructions. High resolution data (resolution 70,000) was acquired by full scan from *m/z* 200–2000 with source voltage of 3500 V electrospray ionisation positive mode (ESI +) or − 3600 V ESI negative mode (ESI −), capillary temperature of 275 °C, and sheath, auxiliary and sweep gas flow rates of 40, 10 and 5 arbitrary units, respectively. Data-dependent MS^2^ data were collected with a mass resolution set to 35,000 recording a mass range of *m/z* 200–2000 and maximum trap fill time of 250 ms (full scan mode) or 120 ms (MS^2^ scan mode). The isolation window of selected MS^1^ scans was ± 1.5 m*/z* with a normalised collision energy of 30 units.

### Liquid chromatography (LC)-mass spectrometry analysis of polar metabolites

Polar metabolites were analysed with an Accela 1250 quaternary UHPLC pump coupled to an Exactive Orbitrap mass spectrometry (Thermo Fisher Scientific, USA). Chromatographic separation was carried out at 25 °C on a SeQuant® ZIC®-pHILIC 5 µm, 2.1 mm × 100 mm column (Merck, Darmstadt, Germany) with the following solvent system: A = 10 mM ammonium formate in water, B = 0.1% formic acid in acetonitrile. A gradient program was used at a flow rate of 250 µL/min: 3–3% A (0.0–1.0 min), 3–30% A (1.0–12.0 min), 30–90% A (12.0–14.5 min), 90% A was maintained for 3.5 min followed by re-equilibration with 3% A for 7 min. An injection volume of 2 µL was used. The electrospray probe was operated unheated at room temperature (20 °C) to avoid degradation of thermally labile compounds. External mass calibration of the Orbitrap prior to sample analysis was performed by flow injection of the calibration mix solution according to the manufacturer’s instruction. High resolution data (resolution 25,000) were acquired by full scan from *m/z* 55 to 1100 with source voltage of 4000 V for ESI + and − 4000 V for ESI − , capillary temperature of 325 °C, and sheath, auxiliary, and sweep gas flow rates of 40, 10, and five arbitrary units, respectively.

### Data processing

Raw data files were converted to mzXML format with MSconvert (v 3.0.1818) and pre-processed. Three lipid datafiles were corrupted and excluded from analysis. Lipid data were preprocessed with the XCMS package (v3.0.2) in the R programming environment (v3.2.2) [40], whereas polar metabolite data were preprocessed with the ADAP algorithm in mzMINE (v2.31) (processing parameters provided in Additional file 2: Table S2) [41, 42]. Features not detected in 100% of the QC samples were excluded. For polar metabolite data, features detected in at least one blank sample before peak filling step were removed. For lipid data, blank features were filtered out based on tstat and p-values (sample vs. blank tstat < 1 or those with tstat > 1 but *p* value ≥ 0.05) generated by the diffreport function from the XCMS package. Manual examination of EIC was conducted to filter out poorly integrated peaks, using build-in function in mzMINE for the polar metabolite data and the EICs generated by the diffreport function in the XCMS package for lipid data. After data cleaning, signal drift and batch effects were corrected by LOESS in the W4M Galaxy environment [43], and features with QC %CV > 30 were removed. Lipid identification was performed using LipidSearch software v4.1.16 on MS^2^ datafiles (Thermo Fisher Scientific, USA) as previously described to generate a MS^2^-annotated lipid ID library and matched against the processed lipid data matrices based on parent mass and retention times [44]. Lipid features without an MS^2^-annotated lipid ID were searched against online database including HMDB (https://www.hmdb.ca/), Metlin (https://metlin.scripps.edu/) and Lipid Maps (https://www.lipidmaps.org/) based on *m/z* with less than 10 ppm mass error. Polar metabolites were annotated using an in-house library based on authentic standards (AgResearch) analysed through HILIC LCMS analysis under conditions identical to the current study. Metabolic features without a hit in the library were searched against online database including HMDB and Metlin based on *m/z* with less than 15 ppm error.

### Statistical analyses

Multivariate analysis using partial least squares-discriminant analysis (PLS-DA) was initially applied to investigate differences in baseline profiles of the two ethic groups (SIMCA version 16, Umeå, Sweden). Logistic regression was subsequently performed to allow for adjustment for age, gender, HDL-C, insulin, FPG, HbA_1c_, BMI and %VAT_TBF_ in identifying discriminatory lipids and metabolites between the two ethnicities (with Caucasian as reference level and Asian Chinese as level 1). To characterise the metabolomic and lipidomic signatures for FPG or %VAT_TBF_ (as the outcome variable), multiple linear regression was performed on every feature adjusting for age, gender and BMI in an ethnicity-specific manner. All univariate statistical analyses were carried out in R (v3.5.1) and subjected to FDR correction (Benjamini Hochberg procedure) [45]. Up till this point the lipid profiles and polar metabolites profiles were analysed separately due to different number of available profiles (3 lipid profiles were corrupted), but the list of associated metabolites (both lipids and polar metabolites) were combined into a single table for each trait of interest for reporting purpose.

The prediction model of FPG state by a panel of metabolites associated with FPG and %VAT_TBF_ in each ethnicity was built using random forest (RF) [46] in MetaboAnalyst v4.0 [47]. In detail, the datamatrix containing intensities of all metabolites associated with FPG and/or %VAT_TBF_ in each ethnicity identified by the aforementioned multiple linear regression, were extracted and imported to MetaboAnalyst for biomarker analysis. Three samples were excluded from the re-stratification due to the absence of their lipidomic profiles. RF analysis was performed on the 100 most important variables ranked by decreases in accuracy through 30 repeats of threefold random sub-sampling cross-validation. Individuals who were predicted as NFG by the metabolomic-based RF model were designated as “mNFG”, whereas those predicted as IFG were designated as “mIFG”. The predicted FPG state was further combined with their actual FPG state as determined by the ADA FPG criteria to create a new stratification system consisted of 4 groups: NFG-mNFG (NFG individuals predicted to be NFG based on their metabolomic profile), NFG-mIFG (NFG individuals predicted to be IFG), IFG-mNFG (IFG individuals predicted to be NFG) and IFG-mIFG (IFG individuals predicted to be IFG). The clinical profile (including HbA_1c_, HOMA2-IR, BMI, age, waist-to-hip ratio, SBP, DBP, ALT, AST, ALP, GGT, total cholesterol, HDL-C, total TG, LDL-C, amylin, C-Peptide, GIP, GLP-1, glucagon, insulin) of the metabolic FPG state and the 4 sub-phenotype were then characterised using multivariate PLS-DA (SIMCA 16, Umetrics) and univariate t-test (R (v3.5.1)).

## Results

Characteristics of the study population are summarised in Table 1. Asian Chinese had higher body weight, DBP, %AAT, %VAT_TBF_, %VAT_AAT_, HbA_1c_, FPG, fasting insulin, total TG, ALT, GGT, amylin, GLP-1 and glucagon, and lower HDL-cholesterol than Caucasians.

**Table 1:** Metabolic risk factors in Caucasian European (N = 158) and Asian Chinese (N = 199) enrolled in the TOFI_Asia Study

	Caucasian European	Asian Chinese	*P value*
n	158	199	
% female	59	54	
Age (year)	41.7 ± 16.1	40.5 ± 13.3	0.47
*Anthropometry*			
Weight (kg)	79.9 ± 15.7	75.6 ± 14.4	0.007
Height (m)	1.72 ± 0.09	1.66 ± 0.08	< 0.0001
BMI (kg/m^2^)	26.9 ± 4.6	27.2 ± 3.9	0.53
Waist circumference (cm)	90.3 ± 14.1	90.0 ± 10.8	0.79
Hip circumference (cm)	101.1 ± 11.8	99.1 ± 10.2	0.07
Systolic blood pressure (mmHg)^a^	120 ± 15	122 ± 17	0.23
Diastolic blood pressure (mmHg)^a^	66 ± 8	69 ± 11	0.002
*Body composition—*^*b*^* DXA*			
Total body fat (TBF) mass (kg)	26.6 ± 11.4	25.5 ± 7.8	0.33
%TBF	33.8 ± 10.2	35.0 ± 7.2	0.21
Abdominal adipose tissue (AAT) mass (kg)	2.3 ± 1.4	2.4 ± 1.0	0.65
AAT (% of abdominal tissue mass)	36.8 ± 14.1	40.8 ± 9.1	0.003
Visceral adipose tissue (VAT) mass (kg)	0.9 ± 0.8	1.0 ± 0.6	0.07
%VAT_TBF_	2.86 ± 2.19	3.73 ± 1.98	0.0001
%VAT_AAT_	32.2 ± 19.9	39.7 ± 16.4	0.0002
*Blood biochemistry*			
HbA_1c_^c^	33.3 ± 3.6	35.8 ± 3.9	< 0.0001
Fasting plasma glucose, FPG (mmol/L)	5.0 ± 0.6	5.3 ± 0.5	< 0.0001
Fasting plasma insulin (pg/ml)	430.4 ± 316.5	570.6 ± 359.9	0.0001
HOMA2-IR	1.8 ± 2.0	1.9 ± 1.2	0.8
HOMA2-β	140.4 ± 122.7	126.2 ± 61.4	0.18
Total cholesterol (mmol/L)^d^	5.0 ± 1.0	4.8 ± 0.9	0.19
Triglycerides (mmol/L), TAG^d^	1.1 ± 0.6	1.4 ± 0.9	0.0001
HDL-Cholesterol (mmol/L)^d^	1.6 ± 0.4	1.4 ± 0.4	< 0.0001
LDL-Cholesterol (mmol/L)^d^	2.9 ± 0.9	2.8 ± 0.8	0.49
Alanine amino transferase, ALT (U/L)^e^	15.8 ± 10.4	19.3 ± 14.0	0.008
Aspartate amino transferase, AST (U/L)^e^	20.9 ± 8.7	19.9 ± 6.4	0.23
Alkaline phosphatase, ALP(U/L)^e^	97.2 ± 27.2	96.2 ± 23.5	0.68
Gamma glutamyl transferase, GGT (U/L)^e^	23.6 ± 18.2	30.2 ± 23.8	0.003
Amylin (pg/ml)	28.8 ± 15.2	33.8 ± 17.4	0.005
C-peptide (pg/ml)	939.7 ± 554.5	935.3 ± 460.7	0.94
Gastric inhibitory peptide, GIP (pg/ml)	72.4 ± 58.2	79.0 ± 48.0	0.25
Glucagon like peptide – 1, GLP-1 (pg/ml)	146.6 ± 62.0	165.4 ± 87.6	0.02
Glucagon (pg/ml)	59.2 ± 35.3	71.1 ± 35.1	0.002

Results are mean ± SD. Numbers were as stated above each column except for bracketed values

^a^Systolic and diastolic blood pressure were measured in 198 Asian Chinese

^b^Body composition was assessed in 157 Caucasian European

^c^HbA1c was measured in 157 Caucasian European

^d^Lipid profile and d liver function were assessed in 198 Asian Chinese. Nonstandard abbreviations: DXA, dual energy X-ray absorptiometry; HbA1c, haemoglobin A1c; HOMA2-IR, Homeostasis Model Assessment of insulin resistance; HOMA2-β: Homeostasis Model Assessment of β-cell function

### Characterisation of fasting plasma profile associated with ethnicity differences

**Fig. 1:**
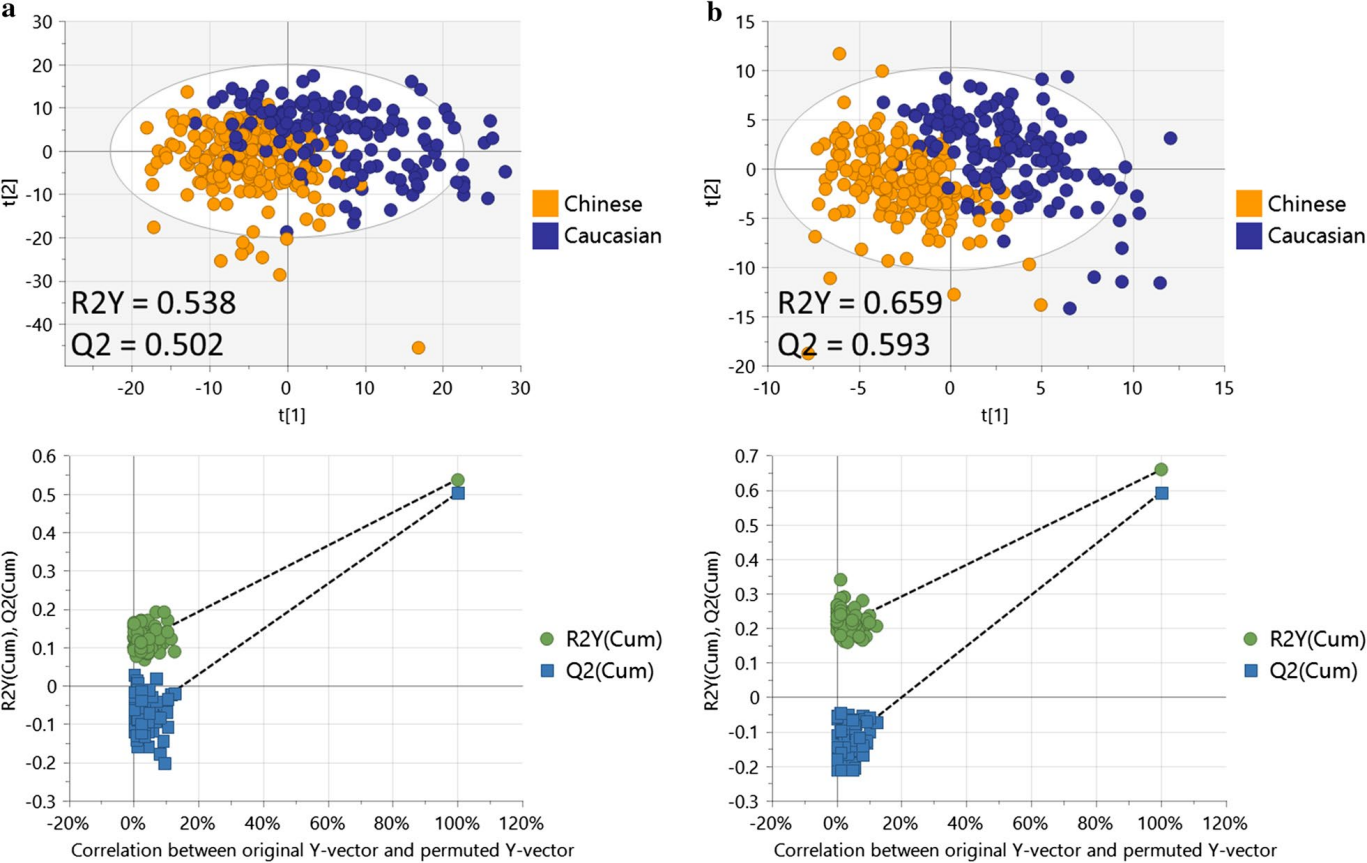
PLS-DA analysis for metabolomics differences between Caucasians and Asian Chinese. PLS-DA score plot (top) and 100 permutation tests (bottom) showing good separation and robust model for: **a** lipid profile and **b** polar metabolite profile, between Asian Chinese and Caucasian

The score plots from the PLS-DA models for both lipid and polar metabolite profiles showed clear and robust separation between Asian Chinese and Caucasian cohorts (Q2 values of 0.502 for lipids and 0.593 for polar metabolites, confirming that the models had acceptable validity), indicating ethnicity influences the fasting plasma metabolome (Fig. 1).

**Fig. 2:**
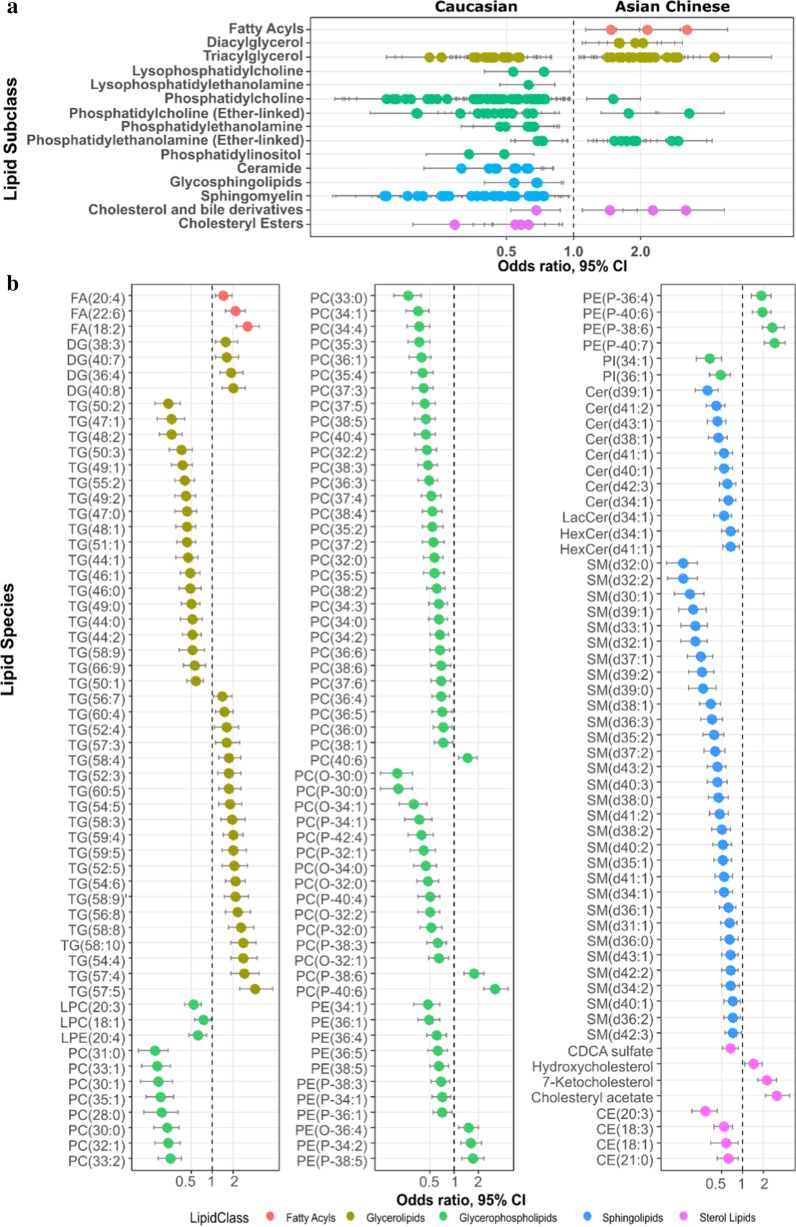
Association of lipid profile with ethnicity. Lipids associated with ethnicity at the level of **a** lipid subclasses and **b** individual lipid species following adjustment for age, gender, HDL-C, Insulin, FPG, HbA1c, BMI and %VAT_TB**F**_. All the displayed lipids have statistically significant *p* value (BH-corrected *p* < 0.05). FA: fatty acid; DG: diacylglycerol; TG: triacylglycerol; LPC: lysophosphatidylcholine; LPE: lysophosphatidylethanolamine; PC: phosphatidylcholine; PC(O-/P-): ether-linked (plasmenyl/plasmanyl) PC; PE: phosphatidylethanolamine; PE(O-/P-): ether-linked (plasmenyl/plasmanyl) PE; PI: phosphatidylinositol; Cer: ceramide; Lac: lactosylceramide; HexCer: glucosylceramide; SM: sphingomyelin; CE: cholesteryl ester; CDCA sulfate: Chenodeoxycholic acid sulfate

Logistic regression was applied to identify metabolites associated with ethnicity independent of potential covariates. Of the 629 lipid features measured by LC–MS, 170 identified lipid species over 15 lipid subclasses in addition to 25 unknowns were significantly associated with ethnicity, independent of potential covariates (BH adjusted *p* < 0.05) (Additional file 2: Table S3 and Fig. 2). Levels of lipid species belonging to lipid classes lysophosphatidylcholine (lysoPC), lysophosphatidylethanolamine (lysoPE), phosphatidylethanolamine (PE), phosphatidylinositol (PI), ceramide (Cer), glycosphingolipids (GCer), sphingomyelin (SM) and cholesteryl ester (CE) were exclusively higher in Caucasians, whereas those belonging to free fatty acid (FFA) and diacylglycerol (DG) classes were exclusively higher in Asian Chinese (Fig. 2a). Although triacylglycerol (TG), phosphatidylcholine (PC), ether-linked PC and ether-linked PE did not display unidirectional association with either ethnic group, some characteristic patterns were observed. TGs containing carbon chains between C12-C18 and with a higher degree of saturation were associated with Caucasians whereas TGs enriched in long and very long chains between C16–C22 and with a higher degree of unsaturation were associated with Asian Chinese (Fig. 2b). The majority of discriminatory PCs and ether-linked PCs were at higher levels in Caucasians with the exception of PC(40:6), PC(p-38:6) and PC(p-40:6) which were higher in Asian Chinese, which again, contained a polyunsaturated fatty acid (PUFA). Ether-linked PEs associated with Asian Chinese also tended to contain more PUFA (Fig. 2b and Additional file 2: Table S3). Despite significantly higher total fasting TG as measured by biochemical assay in Asian Chinese (see Table 1), lipidomics indicates that the molecular makeup of plasma glycerolipid is discriminatory between the two ethnic groups.

**Fig. 3:**
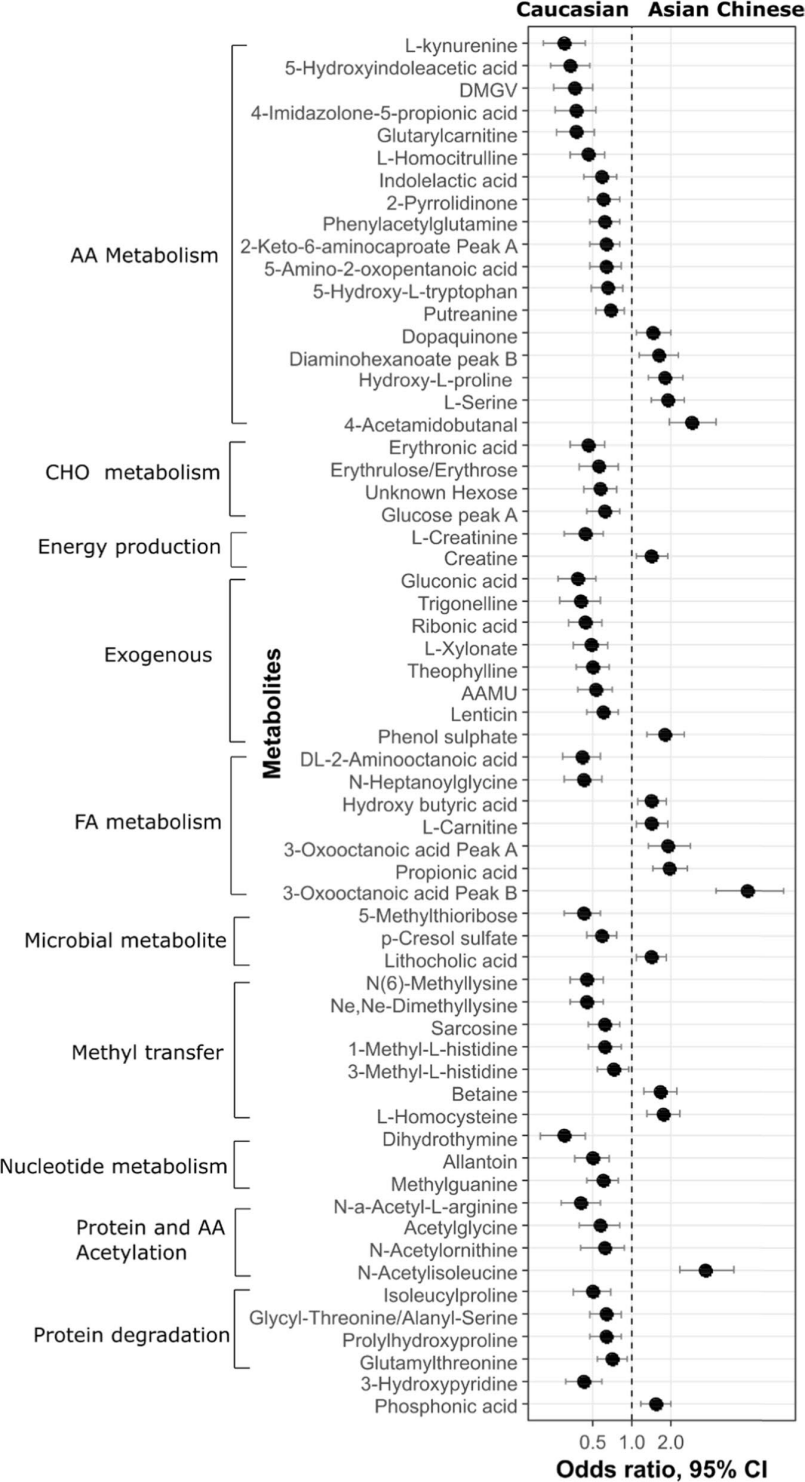
Association of polar metabolite profiles with ethnicity. Differentially expressed polar metabolites in Caucasian and Asian, after adjusting for age, gender, HDL-C, Insulin, FPG, HbA_1c_, BMI and %VAT_TBF_. All the displayed metabolites have statistically significant p value (BH-corrected *p* < 0.05). AAMU: 5-Acetylamino-6-amino-3-methyluracil; DMGV: (alpha-keto-dimethyl-delta-N,N-Dimethylguanidynol) valeric acid

A number of endogenous and exogenous polar metabolites were discriminatory between Caucasians and Asian Chinese (Additional file 2: Table S4). These metabolites spanned a wide range of biological functions and pathways such as AA metabolism, carbohydrate (CHO) metabolism, energy production, fatty acid (FA) metabolism, nucleotide metabolism, protein metabolism and modification and the methyl transfer pathway (Fig. 3).

### Ethnicity-specific metabolomic signatures of FPG and %VAT_TBF_

**Fig. 4:**
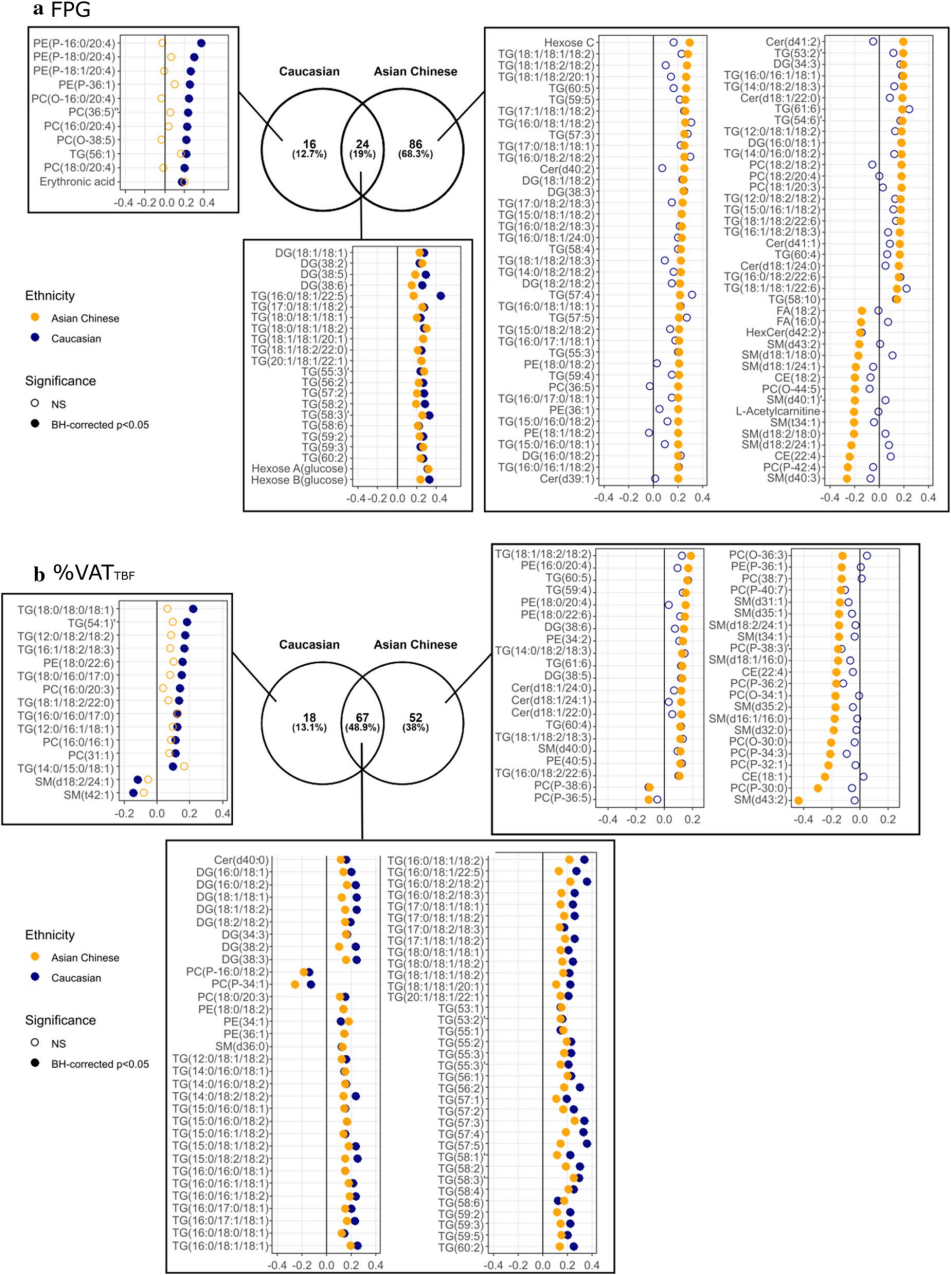
Metabolites significantly associated with FPG and %VAT_TBF_, after adjusting for gender, age and BMI. The Venn diagram showed the number of variables common or unique to Caucasian and Asian Chinese, including both annotated metabolites and unknowns; the adjusted beta-coefficient of every annotated metabolites in Caucasian (blue) and Asian Chinese (orange), were displayed in the coefficient plot

Metabolic features associated with FPG that are common to both ethnic groups or unique to each ethnic group were summarised in Fig. 4a and Additional file 2: Table S5. After adjusting for gender, age and BMI, FPG in Caucasians was associated with 40 non-redundant features yielding 33 identified metabolites, including positive correlations with 4 DG species, 17 TGs, 2 ether-linked PCs, 4 ether-linked PEs, 3 PCs, 2 hexoses (both likely to be glucose peaks as confirmed by internal standard, Additional file 1: Figure S1) and erythronic acid. The phospholipids and ether-linked phospholipids notably contained an arachidonic acyl chain n20:4 (confirmed with MS^2^ spectral data). The strongest marker for FPG other than the MS-measured glucose (in terms of having the lowest raw p-value) in Caucasians was DG(38:5) (adjusted beta-coefficient = 0.29 [95% CI 0.16–0.42], *p* = 3.00E−05). FPG in Asian Chinese was associated with 110 non-redundant features yielding 101 identified metabolites, including positive correlations with 6 ceramides, 10 DGs, 59 TGs, 4 PCs, 3 PEs, 2 hexoses (MS-measured glucose) and an unknown hexose, and negative correlations with 2 CE, 2 FFAs, 8 SMs, 2 ether-linked PCs, HexCer(d42:2) and L-acetylcarnitine. The strongest marker was TG(54:4) (0.28 [95% CI 0.18–0.38], p = 2.02E−07). Only MS-measured glucose, 4 DG species, 16 TG species plus 2 unknowns were common makers for FPG in both ethnic groups (19% overlapped), and many of them contained an oleate moiety (Fig. 4a).

%VAT_TBF_ was associated with many lipid species in both Caucasians and Asian Chinese, whilst no polar metabolites measured by HILIC remained significantly associated with %VAT_TBF_ after multiple testing correction (BH-corrected *p* < 0.05). The %VAT_TBF_-associated lipid features, either unique to each, or common to both ethnic groups, have been provided in Fig. 4b and Additional file 2: Table S6. Independent of gender, age and BMI, %VAT_TB**F**_ in Caucasians was associated with 85 non-redundant features (96% identified), including positive correlations with Cer(d40:0), SM(d36:0), 8 DGs, 4 PCs, 4 PEs and 60 TGs, and negative correlations with 2 ether-linked PCs and 2 SMs. The most strongly associated lipid species based on p-value was TG(56:2) (0.3 [95% CI 0.2–0.4], *p* = 8.25E−09). %VAT_TB**F**_ in Asian Chinese was associated with 119 non-redundant features (92% identified), including positive correlations with PC(38:3), SM(d36:0), SM(d40:0), 4 ceramides, 10 DGs, 8 PEs and 59 TGs, and negative correlations with PC(38:7), PE(P-36:1), 2 CEs, 13 ether-linked PCs and 9 SMs, with TG(58:3) being the most significantly correlated marker (0.25 [95% CI 0.17–0.34], *p* = 2.34E−08). 67 lipid species were common markers for %VAT_TB**F**_ in both ethnic groups, encompassing lipid species of ceramide, SM, PC, PEs, ether-linked PCs, DGs and TGs (48.9% overlapped), and many of them notably contained a linoleate moiety (Fig. 4b).

**Fig. 5:**
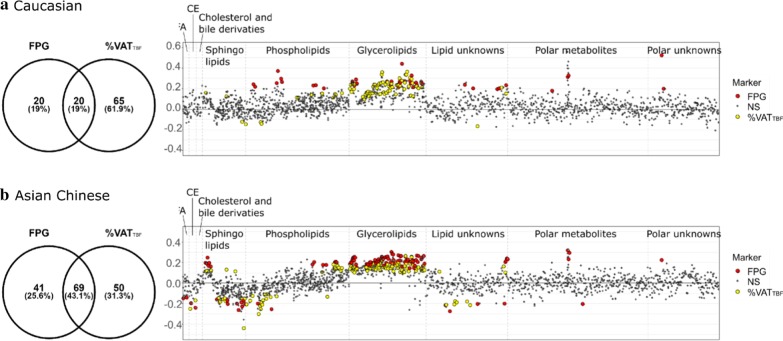
Overlaid metabolomic signature for FPG and %VAT_TBF_ in Caucasians and Asian Chinese. The Venn diagram showed the number of variables common or unique to FPG and %VAT_TBF_, including both annotated metabolites and unknowns; the overlaid coefficient plot on the right displayed adjusted beta-coefficient of all metabolic features, with those being significantly associated with FPG (red) or VA (yellow) highlighted (BH-corrected *p* < 0.05)

Among the 105 variables associated with either FPG or %VAT_TBF_ in Caucasians, 20 were common markers for both FPG and %VAT_TBF_ (19% overlap) after adjusting for age, gender and BMI. These included DG(36:2), DG(38:2) and 17 TG species plus one unknown lipid species (*m*/*z* = 933.8665) (Fig. 5a and Additional file 2: Table S7). Whereas in Asian Chinese, 69 out of 160 variables associated with either trait were common markers for FPG and %VAT_TBF_ (43.9% overlap) independent of age, gender and BMI, including CE(22:4), Cer(d40:1), Cer(d42:1), PE(36:1), PE(36:2), SM(d42:3), SM(d43:2), SM(t34:1) in addition to 10 DG and 50 TG species plus one unknown lipid species (*m*/*z* = 924.8006) (Fig. 5b and Additional file 2: Table S8).

### Predicting FPG state using the metabolomic signature

We have identified individual metabolite significantly associated with FPG and/or %VAT_TB**F**_. Next, a random forest model for predicting IFG state from these metabolites as a set of predictors was constructed. To do so, we combined the list of metabolites associated with FPG or %VAT_TB**F**_ from the previous step but removed the two MS-measured glucose features (i.e. 160–2 = 158 variables for Asian Chinese and 105–2 = 103 variables for Caucasians), and built RF based on 100 top-ranked metabolites for each ethnicity (Additional file 2: Tables S7, S8). The reason for excluding the MS-measured glucose from the re-stratification step is that it is of interest to determine the metabotype using biologically relevant information other than glucose itself, and including glucose may confound the variable selection and sample re-stratification. The RF models achieved a prediction accuracy with an area under receiver operating characteristic (ROC) curve of 0.79 (95% CI 0.704–0.882) in Caucasians and 0.76 (95% CI 0.646–0.847) in Asian Chinese (Additional file 1: Figure S2). These models were then used to define mFPG state, the predicted FPG state based on the metabolomic signature.

### Characterisation of clinical profiles of the metabolic FPG state

After establishing a model to predict metabolic FPG state using the metabolomic signature (i.e. designated as either mNFG or mIFG), participants were stratified into 4 groups for each ethnicity: 2 had predicted FPG states concordant to the actual state (NFG-mNFG, IFG-mIGF), and 2 had a discordant metabolic FPG state from the actual state (NFG-mIFG, IFG-mNFG). 74% of NFG and 77% of IFG individuals had a concordant predicted FPG state (i.e. NFG-mNFG and IFG-mIFG, respectively) in Caucasian; 26% of NFG individuals were predicted to be mIFG (NFG-mIFG) and 23% of IFG individuals were predicted as mNFG (IFG-mNFG). In Asian Chinese, 70% of NFG and 74% of IFG individuals were predicted to have an mNFG and mIFG state, respectively; 30% of NFG individuals had an mIFG state and 26% of IFG individuals had an mNFG state.

**Fig. 6:**
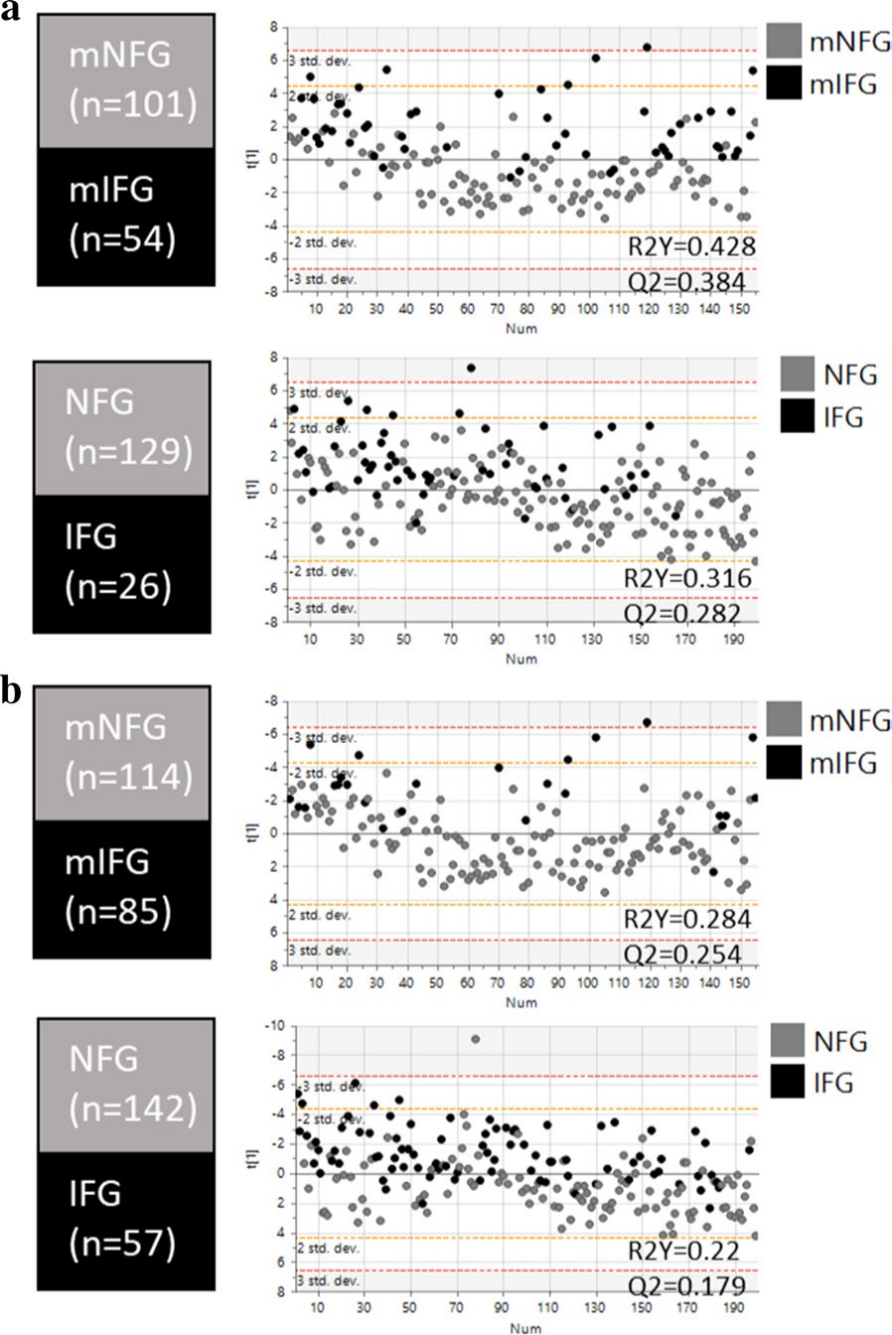
PLS-DA model based on clinical variables associated with cardiometabolic risks as independent variables. Score plots and model performances for PLS-DA models of NFG vs IFG or mNFG vs mIFG in **a** Caucasian and **b** Asian Chinese. Clinical variables HbA_1c_, HOMA2-IR, BMI, age, waist-to-hip ratio, SBP, DBP, ALT, AST, ALP, GGT, total cholesterol, HDL-C, total TG, LDL-C, amylin, C-Peptide, GIP, GLP-1, glucagon, insulin were use as x-variable in the PLS-DA models

**Fig. 7:**
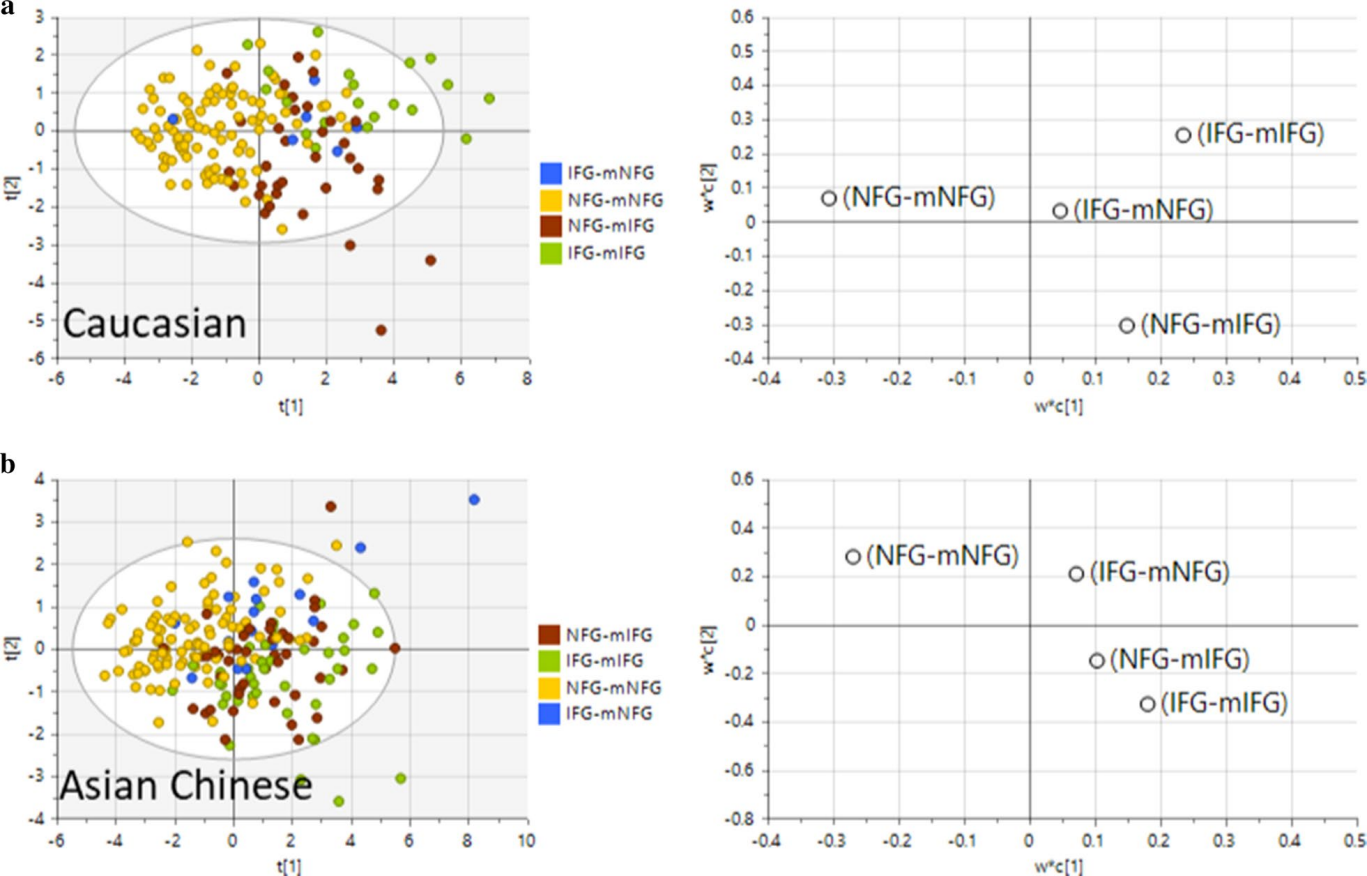
PLS-DA model based on parameters associated with cardiometabolic risks as independent variables. Score plots and loading plots with projected centre from each group for PLS-DA of four level analysis (NFG-mNFG, NFG-mIFG, IFG-mNFG and IFG-mIFG) in **a** Caucasian and **b** Asian Chinese

The clinical and anthropometric profiles between these newly designated groups were then compared using a multivariate PLS-DA or univariate t-test approach. For the PLS-DA model, we first constructed and compared models using 21 clinical and anthropometric measurements as explanatory variables and mNFG vs mIFG as predicted by the metabolomic signature or NFG vs IFG as stratified by ADA FPG criteria (Fig. 6). Both models of mNFG vs mIFG and NFG vs IFG were robust and revealed good separation between sample groups (mNFG vs mIFG model, R2Y = 0.428, Q2 = 0.384 for Caucasians and RY2 = 0.284, Q2 = 0.254 for Asian Chinese; NFG vs IFG model, R2Y = 0.316, Q2 = 0.282 for Caucasians and R2Y = 0.22, Q2 = 0.179 for Asian Chinese), with mNFG vs mIFG model outperforming NFG vs IFG in both ethnicities (Fig. 6). This highlighted the clinical profile was correlated better with the predicted FPG state by metabolomic than the actual FPG state. From the 4-group PLS-DA analysis, component 1 clearly separated NFG-mNFG from all 3 IFG groups (NFG-mIFG, IFG-mNFG and IFG-mIFG) in both ethnic groups, and the centre of NFG-mIFG was projected even closer to IFG-mIFG than to NFG-mNFG (Fig. 7). These results collectively indicated a similar clinical risk profile of individuals delineated by the metabolic FPG state.

**Fig. 8:**
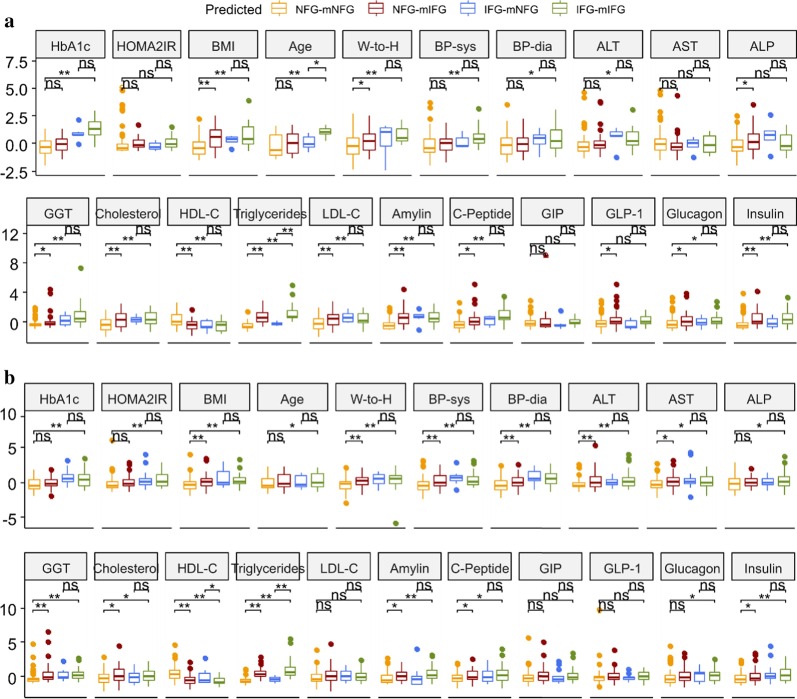
Clinical characterisation the newly assigned metabotype groups. Boxplot showed different levels of parameters associated with cardiometabolic health among healthy (NFG-mNFG), normoglycaemic with “prediabetic” metabolomic signature (NFG-mIFG), impaired fasting glucose with normoglycaemic metabolomic signature (IFG-mNFG), and prediabetic (IFG-mIFG) individual in (a) Caucasian and (b) Asian Chinese. T-test was carried out on each pair of NFG-mNFG vs NFG-mIFG, IFG-mNFG vs IFG-mIFG, NFG-mNFG vs IFG-mIFG (*p < 0.05, **p < 0.01, ns: *p* > 0.05). All measured values were scaled to mean = 0, standard deviation = 1

Comparison of clinical and anthropometric measurements associated with cardiometabolic risk using a univariate approach based on this new stratification revealed a worse cardiometabolic risk profile of NFG-mIFG individuals compared to the NFG-mNFG, despite all currently having a ‘normal’ fasting glucose level. This included higher adiposity-related parameters (BMI, waist-to-hip ratio), liver enzyme (ALP, GGT), total cholesterol, total TG, LDL-C, glucoregulatory hormones (amylin, C-peptide, GLP-1, glucagon, insulin), and lower HDL-C in Caucasians (*p* < 0.05) (Fig. 8a). In Asian Chinese, NFG-mIFG individuals were characterised by higher blood pressure (SBP, DBP), adiposity-related parameters (BMI, waist-to-hip ratio), liver enzymes (ALT, AST, GGT), total cholesterol, total TG, glucoregulatory hormones (amylin, C-peptide, insulin), and lower HDL-C than NFG-mNFG individuals (*p* < 0.05) (Fig. 8b). Conversely, Caucasian IFG-mNFG individuals tended to be younger and had lower total TG, and Asian Chinese IFG-mNFG individuals had lower total TG and higher HDL-C than their IFG-mIFG counterparts (p < 0.05).

## Discussion

T2D is a progressive disease that may be prevented if at-risk individuals can be identified before disease onset and managed through lifestyle and dietary intervention [48]. As such, prediabetes has attracted considerable attention as it represents a stage where individuals have a suboptimal glycaemic profile and a higher risk of proceeding to T2D onset [49]. Metabolite markers identified using metabolomics may provide insights into the metabolic perturbation contributing to T2D development and help to identify at-risk individuals. Notably most metabolomics studies screening for biomarkers have focused on a single population, however ethnicity is also a key risk factor for T2D that requires investigation. The greater susceptibility of certain populations to T2D with higher propensity of visceral fat deposition suggests metabolic alterations contributing to T2D development are likely to differ between ethnicities [50]. Our study provides compelling evidence for a highly discriminatory fasting plasma metabolome between Asian Chinese and Caucasians. The major differences included a number of AA-related metabolites involved in tryptophan and histidine metabolism, methyl transfer pathway, sugar derivatives, gut microbial metabolites and exogenous compounds, as well as lipid species encompassing 15 lipid subclasses. These results highlighted the need for investigating metabolic alterations and biomarkers associated with risk for T2D development in each ethnicity separately. Subsequently, the ethnicity-stratified analysis showed that FPG was associated with a wide range of lipid species and fewer polar metabolites in both ethnic groups, emphasising a prominent shift in lipid metabolism associated with impaired fasting glucose. Common markers for FPG across both ethnic groups included DG and TG species and the MS-measured glucose. The association between elevated glycerolipids and development of T2D has been well documented, and our results are in line with other metabolomics studies [51–53]. FPG was additionally associated with 3 lipid classes and erythronic acid in Caucasian, and 8 lipid classes, acetylcarnitine and an unknown hexose in Asian Chinese. These associations were unique to each ethnic group and might underlie different metabolic perturbation contributing to development of dysglycaemia. Notably, more lipid markers remained significantly associated with FPG in Asian Chinese after adjustment for age, gender and BMI than in Caucasians, and a larger portion of markers for FPG overlapped with those for %VAT_TB**F**_ in Asian Chinese (43.9% overlap) than in Caucasians (19% overlap), suggesting visceral adiposity was more closely related to the development of dysglycaemia in Asian Chinese independent of total adiposity.

With limited studies investigating metabolic alterations associate with VAT deposition, we here provided evidence demonstrating visceral adiposity profoundly affected lipid metabolism whilst changes of polar metabolites were only weakly associated (i.e. no metabolites passed the significance level after multiple testing correction). 67 lipid species belonging to 7 classes were common %VAT_TB**F**_ markers to both ethnic groups, and %VAT_TB**F**_ was specifically associated with 2 additional lipid classes in Asian Chinese. Both ethnic groups had a TG species (TG(58:2) for Caucasians and TG(58:3) for Asian Chinese) as the most significant marker (i.e. lowest p-value) correlated with %VAT_TB**F**_. The similar pattern of lipid profiles associated with %VAT_TB**F**_ across the 2 ethnic groups suggested a homogenous metabolic adaptation to visceral adiposity irrespective of ethnic background.

Dysregulation of lipid metabolism is a characteristic of T2D and suggested to be a metabolic event prior to the onset of dysglycaemia [54]. Meikle et al. have comprehensively measured the lipid profile associated with prevalent prediabetes and T2D [51]. Many of their observations were replicated in our Asian Chinese group, including association with glycerolipids, ceramides, PEs and ether-linked PCs. Ceramides have been implicated in insulin resistance (IR) as mediators of lipotoxocity and strong associations between high levels of plasma ceramides and reduced insulin sensitivity, prediabetes (glucose intolerance as determined by oral glucose tolerance test) and T2D have been previously reported [55]. In addition, we detected an inverse association between levels of FPG and SMs and hexosylceramide. In a previous cross-sectional study comprising 111 Asian participants, levels of SMs were also markedly lower in IFG/T2D individuals than healthy controls [56]. SMs also inversely correlated with T2D risk in prospective studies including the European Prospective Investigation into Cancer and Nutrition (EPIC)-Potsdam and PREDIMED trials [53, 57]. The positive correlation of ceramides concomitant with negative correlation of SM species and hexosylceramide with FPG observed in the present study reinforced a role of altered sphingolipid metabolism in T2D pathogenesis. Interestingly, %VAT_TB**F**_ also correlated with several ceramide species and dihydroceramide Cer(d40:0), and negatively correlated with SM species in Asian Chinese, highlighting a link between visceral adiposity and altered sphingolipid metabolism. Remarkably, all ceramide species correlated with %VAT_TB**F**_ observed in our study contained a very long acyl chain (C22:0, C24:0 and C24:1), all of which are likely to be products of CerS2, a ceramide synthase showing substrate specificity for longer acyl chain species which is predominantly expressed in liver [58, 59]. Collectively, visceral adiposity might be associated with an altered sphingolipid metabolism that might partly contribute to hepatic IR and confer early dysglycaemia in Asian Chinese. An altered sphingolipid profile was also associated with visceral adiposity but not FPG in Caucasians, suggesting an altered sphingolipid metabolism was a consistent trait for increased VAT deposition across both ethnicities but not directly implicated in early dysglycaemia in Caucasians.

Our results confirmed a metabolic shift in the phospholipid profile implicated in T2D development [60]. However, the molecular makeup segregated between the two ethnic groups. FPG positively correlated with a number of PCs in both ethnic groups, and additionally correlated with PEs in Asian Chinese. We also observed elevated PCs and PEs associated with %VAT_TB**F**_ in both ethnic groups. The association between PEs and prediabetes/T2D was highlighted in Meikle’s study as well as in another study conducted in a Chinese cohort [51, 61]. Both PEs and PCs were also reported to be associated with incident T2D [53, 57]. A concordant alteration of phospholipids and glycerolipids has been suggested to relate to hypertriglyceridaemia and increased very low-density lipoprotein (VLDL) production [62].

We have intriguingly observed an inverse correlation of FA(16:0) and FA(18:2) with FPG in Asian Chinese, as opposed to several studies reporting associations of elevated FFA with IR and development of T2D [56, 63, 64]. One explanation might be an increased uptake and utilisation of FFA by the liver to fuel TG re-esterification and VLDL secretion [65, 66]. In fact, a recent study has shown a U-shaped instead of linear relationship between plasma FFA and insulin resistance in Chinese, and this trend was more prominent in non-obese individuals [67]. As elevated plasma FFA is a characteristic of adipose tissue IR [68], our data did not support adipose tissue IR as a primary factor associated with early dysglycaemia in our Asian Chinese cohort; instead, hepatic IR or hyperinsulinaemia leading to increased hepatic uptake and utilisation of FFA might explain such an inverse association. In addition, we observed an inverse correlation between acetylcarntine and FPG in Asian Chinese. Acetylcarnitine is the product of complete beta-oxidation as well as a substrate for lipogenesis, and an inverse correlation may reflect impairment in complete FAO or an increased utilisation for lipogenesis. Our method did not detect other acylcarnitines, thus it is difficult to conclude whether such an inverse correlation was due to incomplete FAO. Profiling of acylcarnitines with a targeted approach in the future may help to explain this association.

We observed a negative correlation between FPG and an ether-linked PC in Asian Chinese, consistent with other reports of an inverse association of this lipid class with prevalent and incident T2D, obesity and IR [51, 53, 57, 69]. In addition, an inverse association between ether-linked PC and %VAT_TB**F**_ was observed in both ethnic groups, in good agreement with a previous finding of an ether lipid signature characteristic for VAT deposition [31]. Little is known about the biological role of ether-linked PC despite accumulating evidence showing its association with metabolic health. It might act as a free radical scavenger and protect against LDL oxidation [70, 71]. In contrast to Asian Chinese, a mixture of ether-linked PC and ether-linked PE positively correlated with FPG in Caucasians, all of which contained an arachidonic acyl chain. Ether-linked phospholipids are also sources of arachidonic acid and arachidonic acid is a precursor of the production of pro-inflammatory eicosanoids [70, 72]. Our result suggested an elevated FPG in Caucasians was concurrent with an increased lipid reservoir for secondary messengers with pro-inflammatory potential, which might accelerate insulin resistance and the development of T2D.

Contrary to several studies demonstrating a positive association between CE and prediabetes/T2D, we have unexpectedly found an inverse association. However, the PREDIMED trial has reported an inverse association between CEs and risks of T2D and CVD [53, 73], and the authors linked this observation to the “atherogenic lipoprotein phenotype” (characterised by increasing atypically small and dense LDL, usually accompanied by elevated plasma TG and reduced HDL [74]). The small and dense LDL particles are packed with TG instead of CEs [75], and are suggested to be preferentially cleared from plasma via a receptor-independent pathway and therefore exhibit enhanced atherogenic potential [76]. A previous intervention study has shown a high CHO diet is a primary dietary factor driving the atherogenic lipoprotein pattern whereas a restricted CHO diet attenuated this expression [77]. Without dietary record in the present study it is hard to comment beyond this point; but considering the cultural background of Asian Chinese tending to consume large amount of refined grain (e.g. rice), our interesting finding prompts the hypothesis that the association between CE and risk factors for T2D development may be interrelated with diet.

In addition to the MS-measured glucose itself, FPG was correlated with erythronic acid in Caucasians and an unknown hexose in Asian Chinese. Erythronic acid is a novel hitherto-unreported marker that will require further validation, whereas increased hexose (other than glucose) associated with prediabetes and T2D was in line with the others [56, 78].

Taking the metabolomic signature jointly determined by %VAT_TB**F**_—and FPG-associated metabolomic profiles, we were able to create a novel stratification with a subset of normoglycaemic individuals whose metabolomic signature resembled that of prediabetes (termed NFG-mIFG). In both ethnicities they were characterised by higher BMI and W-to-H ratio, and worse lipid (higher total cholesterol and TG, lower HDL-C), liver enzyme and hormone (amylin, C-peptide and insulin) profiles compared to NFG-mNFG counterparts. Of particular interest were the higher liver enzymes, which are indicators of inflamed or damaged liver cells. The liver is a key organ in the regulation of energy homeostasis and metabolism, and is proposed as the primary site affected by excess VAT deposition [14]. Hepatic IR may result from release by VAT into the portal vein of lipolytic products such as FFA and adipokines, as well as inflammatory cytokines by infiltrated macrophages [79]. Importantly, the multivariate statistical modelling revealed improved discrimination between mNFG and mIFG metabotypes than clinical NFG and IFG classification, suggesting a metabolomics-derived signature was more reflective of the integrated changes across a broad range of cardiometabolic risk factors. NFG-mIFG individuals maybe more susceptible to rapid development of T2D than NFG-mNFG despite current normoglycaemia. Validation of these predictions are required through a longitudinal follow up study, or retrospectively on already existing longitudinal public datasets.

One strength of our study is the simultaneous measurement of metabolomic profiles from two co-located ethnic groups with the same extraction protocol and analytical platform, allowing direct comparison whilst minimising environmental confounding factors to inform ethnicity-specific changes in metabolism associated with T2D development. The use of unbiased, highly sensitive and complementary methods enabled a more holistic view of metabolic perturbation associated with increased visceral adiposity and FPG. In addition, we have utilised the metabolomic signature characteristics to develop a possible risk prediction of T2D development, identifying individuals with a worse cardiometabolic profile despite having normoglycaemia. Limitations include the nature of cross-sectional studies which precludes conclusion on causality and requires follow-up to confirm our findings. In spite of a wide range of metabolites and lipids measured by untargeted metabolomics, this approach may not be optimal if a particular class of metabolites/lipids is of interest, hence it is inevitable that some of the findings by others using metabolite class-optimised targeted approaches such as the analysis for acylcarnitines were not observed in our study.

## Conclusions

Our study has revealed a broad spectrum of lipid species associated with FPG and %VAT_TB**F**_ in both Caucasian and Asian Chinese independent of age, gender and BMI. We have shown plasma metabolomic profile to be profoundly influenced by ethnicity, and are the first to compare an ethnicity-specific signature for T2D risk factors including FPG and visceral adiposity. A similar signature for %VAT_TB**F**_ across both ethnicities but a very different signature for FPG observed in the present study highlighted homogeneous metabolic adaptations and alterations in response to VAT deposition, yet a distinct underlying pathogenesis of dysglycaemia. Predictive modelling using the joint metabolomic signature of FPG and %VAT_TB**F**_ has identified a subset of individuals with worse cardiometabolic risk despite current healthy normoglycaemia. This novel approach of re-stratification using the metabolomic signature aids early identification of those at-risk of T2D. This modelling approach could be applied to a wide range of diseases.

## Supplementary information

**Additional file 1**. **Figure S1**: Annotation of glucose peak measured by ESI+ (a) and ESI- (b).** Figure S2**: Receiver operating characteristic (ROC) curve of RF performed 100 most important variables among the pooled list of those associated with FPG or %VATTBF in (a) Caucasian and (b) Asian Chinese.

**Additional file 2**. **Table S1**: Internal standard added to extraction solvent for monitoring signal variation during instrumental analysis.** Table S2**: Data pre-processing parameters for lipidomic profile (XCMS) and HILIC profile (ADAP).** Table S3**: Differentially expressed lipid species between Caucasian and Asian Chinese.** Table S4**: Differentially expressed metabolites between Caucasian and Asian Chinese.** Table S5**: Metabolites associated with FPG in Caucasian and/or Asian Chinese analysed by multiple linear regression adjusted for gender, age and BMI (BH-corrected p<0.05).** Table S6**: Metabolites associated with %VATTBF in Caucasian and/or Asian Chinese analysed by multiple linear regression adjusted for gender, age and BMI (BH-corrected p<0.05).** Table S7**: Metabolites associated with FPG and/or %VATTBF in Caucasian analysed by multiple linear regression adjusted for gender, age and BMI (BH-corrected p<0.05). The list of metabolites is ordered by the variable importance in ramdon forest, and the top 100 variables (from unknown X160_HP_241.0719_12.18 to unknown LP_802.5932_348.16) were selected for sample restratification.** Table S8**: Metabolites associated with FPG and/or %VATTBF in Asian Chinese analysed by multiple linear regression adjusted for gender, age and BMI (BH-corrected p<0.05). The list of metabolites is ordered by the variable importance in random forest, and the top 100 variables (from unknown X160_HP_241.0719_12.18 to PE(P-36:1)) were selected for sample restratification.

## Data Availability

The datasets used and/or analysed during the current study are available from the corresponding author on reasonable request.

## Abbreviations

AA: Amino acids
AAT: Abdominal adipose tissue
ADA: American Diabetes Association
ALP: Alkaline phosphatase
ALT: Alanine transaminase
AST: Aspartate transaminase
BCAA: Branched chain amino acids
BMI: Body mass index
Cer: Ceramide
CHO: Carbohydrate
DBP: Diastolic blood pressure
DG: Diglyceride
DXA: Dual energy X-ray absorptiometry
ESI: Electrospray ionisation
FAO: Fatty acid oxidation
FFA: Free fatty acid
FPG: Fasting plasma glucose
Gcer: Glycosphingolipid
GGT: Gamma-glutamyltransferase
GIP: Gastric inhibitory polypeptide
GLP-1: Glucagon-like peptide-1
HbA1c: Hemoglobin A1C
HDL-C: High density lipoprotein-cholesterol
HOMA2-IR: Homeostasis model assessment of insulin resistance
HOMA2-β: Homeostasis Model Assessment of β-cell function
IFG: Impaired fasting glucose
IR: Insulin resistance
LCAC: Long-chain acylcarnitine
LC–MS: Liquid chromatography–mass spectrometry
MCAC: Medium-chain acylcarnitine
mIFG: Metabolomics-predicted IFG state
mNFG: Metabolomics-predicted NFG state
NFG: Normal fasting glucose
PC: Phosphatidylcholine
PE: Phosphatidylethnolamine
PI: Phosphatidylinositol
PLS-DA: Partial least squares-discriminant analysis
PUFA: Polyunsaturated fatty acid
RF: Random forest
SBP: Systolic blood pressure
SM: Sphingomyelin
T2D: Type 2 diabetes
TBF: Total body fat
TCA: Citric acid cycle
TG: Triglyceride
TOFI: Thin-on-the-Outside-Fat-on-the-Inside
VAT: Visceral adipose tissue
W-to-H ratio: Waist-to-hip ratio
