# Supplementary material for: Metabolomic signatures for visceral adiposity and dysglycaemia in Asian Chinese and Caucasian European adults: the cross-sectional TOFI_Asia study
Source: Nutr Metab (Lond). 2020 Nov 16;17:95. doi: 10.1186/s12986-020-00518-z (PMC7667766; doi:10.1186/s12986-020-00518-z)
Supplement: Supplementary file 1 — Additional file 1. Figure S1: Annotation of glucose peak measured by ESI+ (a) and ESI- (b). Figure S2: Receiver operating characteristic (ROC) curve of RF performed 100 most important variables among the pooled list of those associated with FPG or %VATTBF in (a) Caucasian and (b) Asian Chinese. [file 12986_2020_518_MOESM1_ESM.docx]

# Supplementary Figure 1


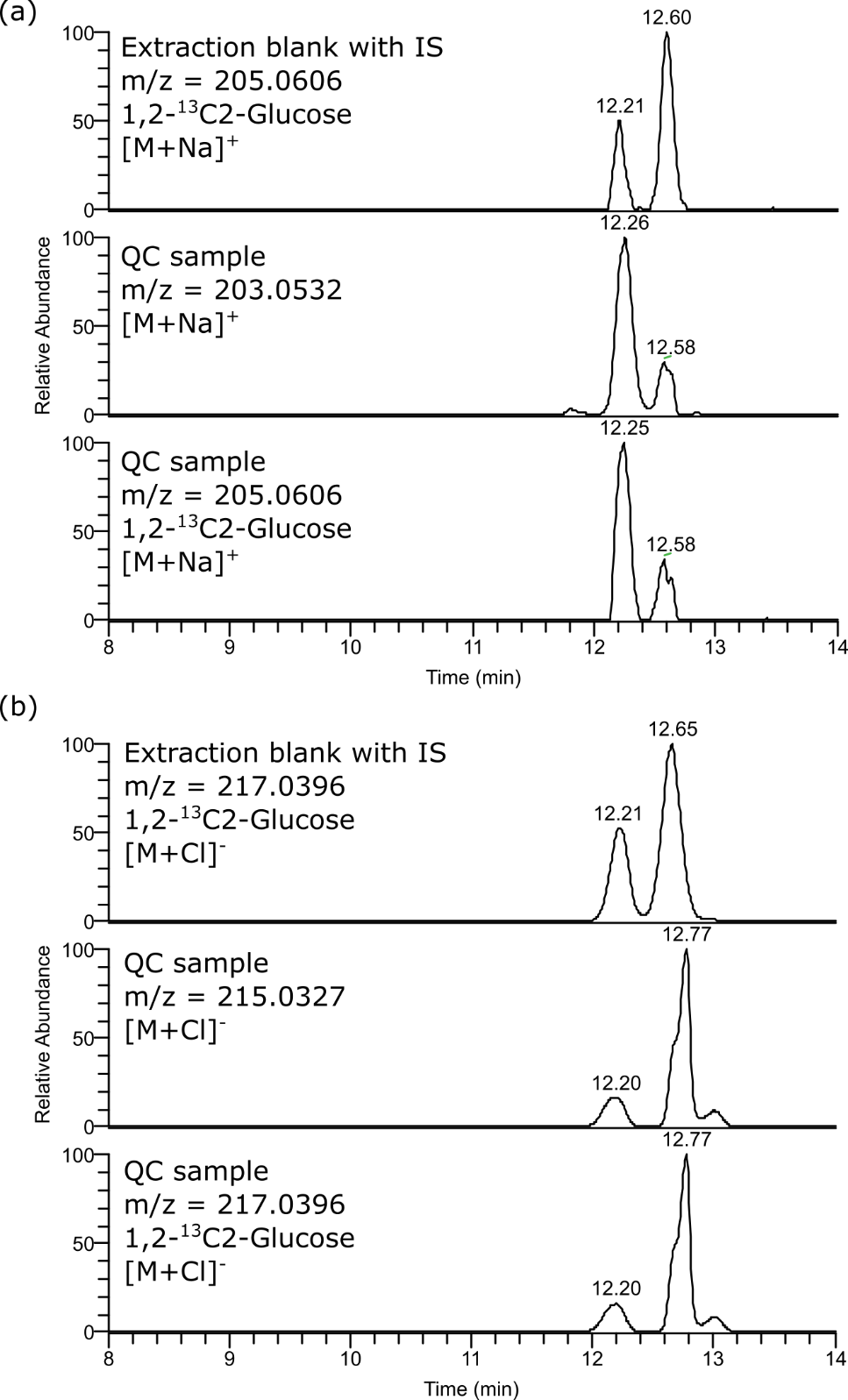


**Figure S1**: Annotation of glucose peak measured by ESI+ (a) and ESI- (b). Two peaks were eluted at 12.2 and 12.6 minutes respectively, confirmed by internal standard 1,2-^13^C2-Glucose added in extraction solvent.

# Supplementary Figure 2


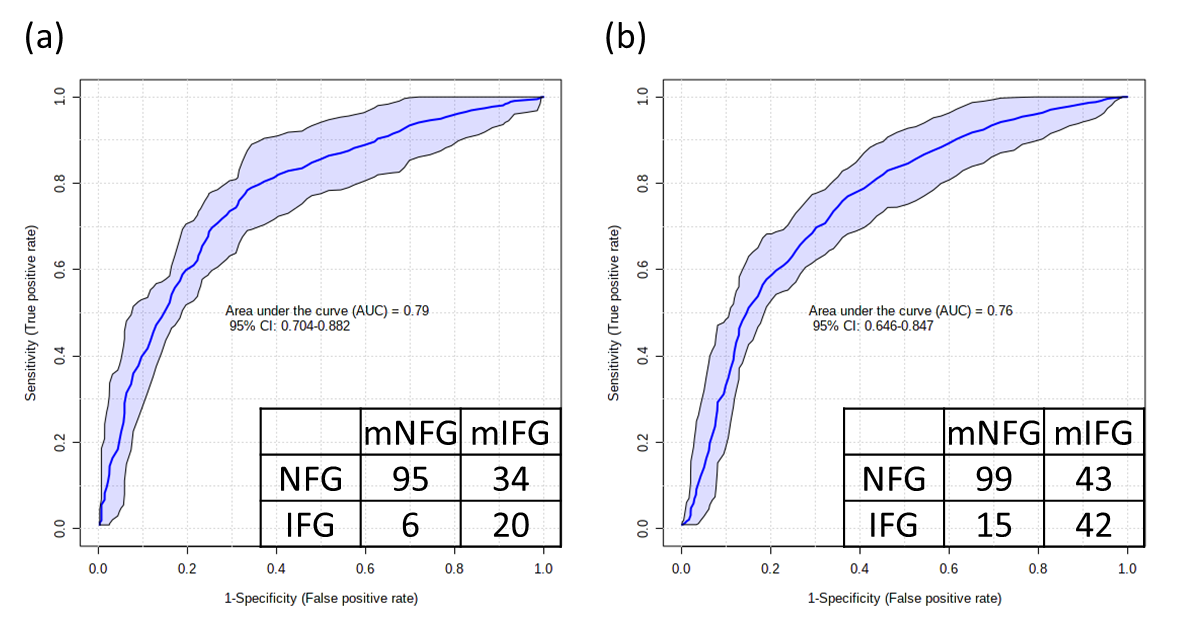


**Figure S2**: Receiver operating characteristic (ROC) curve of RF performed 100 most important variables among the pooled list of those associated with FPG or %VAT_TBF_ in (a) Caucasian and (b) Asian Chinese.
